# Gas Sensing Properties of SnO_2_-Pd Nanoparticles Thick Film by Applying In Situ Synthesis-Loading Method

**DOI:** 10.3390/s23052404

**Published:** 2023-02-21

**Authors:** Jeong In Han, Sung-Jei Hong

**Affiliations:** 1 Department of Chemical and Biochemical Engineering, Dongguk University-Seoul, Seoul 04620, Republic of Korea; 2 Display Research Center, Korea Electronics Technology Institute, Seongnam 13509, Gyeonggi, Republic of Korea

**Keywords:** in situ synthesis-loading, low temperature, SnO_2_-Pd nanoparticles(NPs), gas sensitive thick film, gas sensitivity

## Abstract

In this study, SnO_2_-Pd nanoparticles(NPs) were made with an in situ synthesis-loading method. The in situ method is to simultaneously load a catalytic element during the procedure to synthesize SnO_2_ NPs. SnO_2_-Pd NPs were synthesized by using the in situ method and were heat-treated at 300 °C. As a result, tetragonal structured SnO_2_-Pd NPs, having an ultrafine size of less than 10 nm and a uniformly distributed Pd catalyst in the SnO_2_ lattice, were well made and a gas sensitive thick film with a thickness of c.a. 40 μm was well fabricated by using the NPs. Gas sensing characterization for CH_4_ gas indicated that the gas sensitivity, R_3500_/R_1000_, of the thick film consistent with SnO_2_-Pd NPs synthesized with the in situ synthesis-loading method, followed by heat-treatment at 500 °C, was enhanced to 0.59. Therefore, the in situ synthesis-loading method is available for synthesis of SnO_2_-Pd NPs for gas sensitive thick film.

## 1. Introduction

Semiconductor type tin oxide nanoparticles (SnO_2_ NPs) have been widely used for various applications in gas sensing for a variety of gases [1,2,3,4,5,6,7,8,9,10,11,12]. Especially, SnO_2_ NPs are the most reliable material to detect methane (CH_4_) gas, which is a main component of natural gas, colorless, odorless, widely used in various industries and people’s daily life, but may form an explosive mixture with ambient air [13]. Recently, attempts have been made to adopt this method to inorganic photo-resistance for extreme-ultraviolet (EUV) lithography [14]. The sensing mechanism is based on the adsorption reactions of gaseous species [15]. In order to cause a reaction of the ultrafine particle with the gas at lower temperature, a noble metal such as palladium (Pd) is applied as catalytic element [16,17]. The catalyst attracts the target gas and, therefore, the catalytic elements should be uniformly distributed on the surface of the SnO_2_ NPs, like nano-hetero-structured materials [18,19], to enhance the sensing properties. In the current synthetic method, however, the uniformity of the catalyst distribution is not guaranteed owing to the limitation of the current method, i.e., the catalytic element is apt to be locally segregated on some part of the SnO_2_ NPs because the catalyst is loaded on the surface of the solid phase NPs. In addition, chemical compounds used in the current catalyst loading method include a chloride component [20,21]. To remove the chloride, all the nano-sized particles as well as the chloride should be heated above 700 °C [22,23,24]. The particle coarsening occurs during the heat-treatment and, as a result, size control of the nano-dimensional particle via the current process becomes very difficult. These issues generate unstable sensitivity during long-term operation due to the growth of SnO_2_ NPs at the catalyst poor region. In our research group, we studied the synthesis of SnO_2_-Pd NPs by using Pd acetate instead of Pd chloride, and a low temperature catalyst adding (LTCA) method was invented [23,24]. In the literature, Pd acetate was used for adding the Pd catalyst to the surface of SnO_2_ NPs. Because acetate was easily burnt out at 300 °C, SnO_2_-Pd NPs were better made at a low temperature. However, uniformity of Pd distribution is still as issue in the SnO_2_ NPs used as gas sensor, and the number of process steps has to decrease for cost reasons.

Thus, in this study, uniform dispersion of Pd catalysts in SnO_2_ NPs was attempted. The Pd catalyst was loaded into the SnO_2_ NPs during the synthesis. We named the synthetic method in situ synthesis-loading, i.e., an in situ method to load the liquid phase catalyst to the liquid phase sensing materials. Thus, synthesis-loading is performed simultaneously to advance the uniformity of catalyst distribution. As experienced in a previous study, lowering the heat-treatment temperature is important to decrease particle size [23,24]. The synthetic method proposed in this study has advantages in decreasing the synthetic process steps compared to current methods of synthesizing SnO_2_-Pd NPs, and the Pd catalyst can be uniformly distributed because it is simultaneously loaded in and on the SnO_2_ lattice, leading to fast response/recovery rate and long-term stability [23,24]. Considering thermal behaviors, the raw material was determined to lower the heat-treatment temperature to less than 300 °C. The SnO_2_-Pd NPs synthesized with the in situ method was compared with that with the current synthetic method. Gas sensitive thick films were also fabricated to evaluate the sensing properties of the SnO_2_-Pd NPs synthesized with the in situ method.

## 2. Materials and Methods (Figure 1)

### 2.1. Preparation and Evaluation of SnO_2_-Pd NPs

As raw materials, Sn(II) acetate and Pd(II) acetate were used. Sn acetate was dissolved in acetone and Pd acetate was dissolved in the same solvent at a concentration of 3 wt% in SnO_2_ NPs. Then, we mixed the two solutions in which Sn(II) acetate and Pd(II) acetate had been dissolved, respectively, and the mixed solution was stirred while evaporating the acetone solvent. The remaining components were then dried at 80 °C in a convection oven to erase the residual solvent, leading to a SnO_2_-Pd NPs precursor. The precursor was heat-treated at 300 °C to crystallize the SnO_2_-Pd NPs. For comparison, we prepared SnO_2_-Pd NPs by using the current method, i.e., a Pd catalyst was loaded onto the surface of SnO_2_ NPs that had already been crystallized to oxide form.

**Figure 1:**
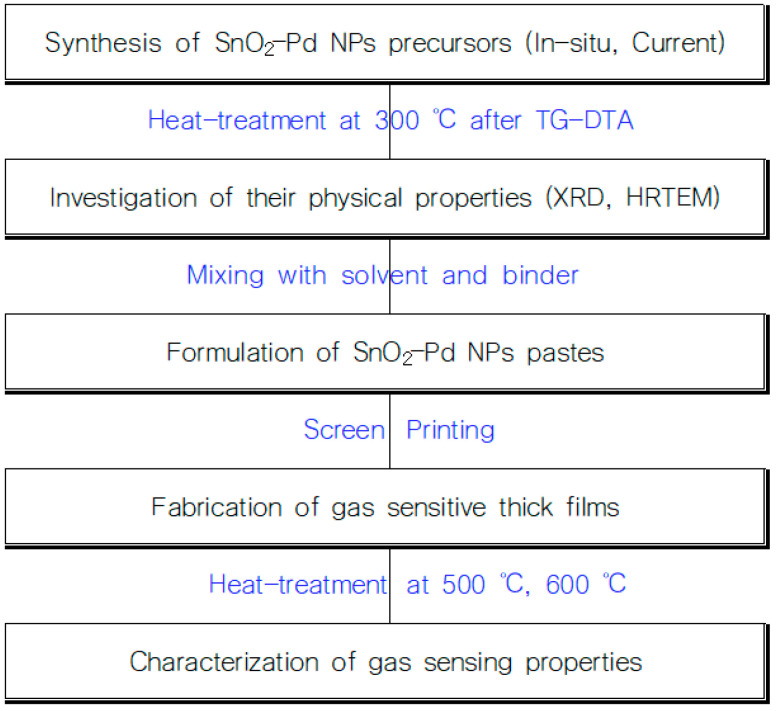
Example diagram of the gas sensors.

To determine whether the precursor is appropriate to the low heat-treatment temperature of 300 °C, thermal behavior was analyzed by using thermogravimetric-differential thermal analysis (TG-DTA) with temperature ranging from 25 °C to 1000 °C at a rate of 10 °C/min. Physical properties of the SnO_2_-Pd NPs, after heat-treatment, were analyzed by high resolution transmission electron microscope (HRTEM, JEOL 300 kV) with an energy dispersion Spectroscope (EDS), X-ray diffractometer (XRD, Rigaku Rotaflex D/MAX System), Brunauer, Emmett & Teller (BET) surface area analyzer and X-ray photoelectron spectroscope (XPS, ESCALAB 210).

### 2.2. Preparation and Evaluation of SnO_2_-Pd NPs Gas Sensitive Thick Films

By using the SnO_2_-Pd NPs, gas sensitive pastes were formulated with and organic based binder, along with butyl carbitol acetate (BCA) solvent. Then, by using the SnO_2_-Pd NPs pastes, gas sensitive thick films were screen-printed on the alumina substrate of size and thickness 2 × 2 mm^2^ and 0.25 mm, respectively. Next, to burn out the solvent and binder in the thick films, the thick film layers were heat-treated at 500 °C and 600 °C, respectively. Finally, their gas sensitivities were evaluated with 500~10,000 ppm methane (CH_4_) gas while aging at 400 °C, which is the minimum operating temperature at which SnO_2_-Pd NPs react with the gas [22]. The gas sensor measurement system consists of a temperature & humidity chamber, a mass flow controller(MFC), a power supply and a computer for measuring program execution and acquisition/storage of measured data. The concentration of CH_4_ gas was controlled by the gas flow rate setting value of MFC calculated for the volume of the chamber and the injection time, and the characteristics of the gas sensor were measured for the preset concentration. When the gas was injected into the chamber, a fan was operated so that the gas was evenly distributed in the chamber. The sensitivity was determined by the ratio of resistances measured at the CH_4_ concentration of 3500 ppm divided by 1000 ppm, i.e., R_3500_/R_1000_. Since the resistance of the gas sensitive layer tends to decrease along with the increasing concentration of the CH_4_ gas, the lower value of R_3500_/R_1000_ indicates the enhanced gas sensitivity.

## 3. Results

### 3.1. Physical Properties of SnO_2_-Pd NPs

**Figure 2:**
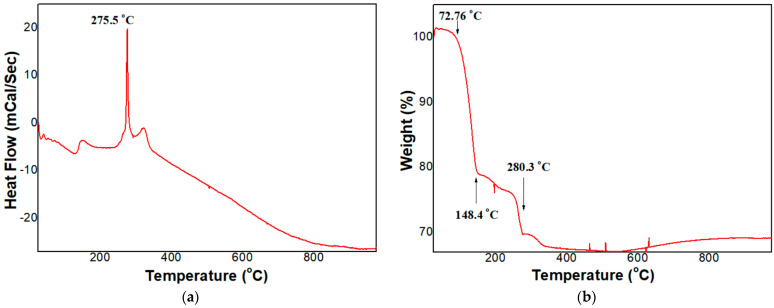
(**a**) Differential thermal analysis and (**b**) Thermogravimetric analysis of Sn(II) acetate.

Thermal behavior of the Sn(II) acetate is shown in Figure 2a,b. Differential thermal behavior, in Figure 2a, reveals that the change in heat flow of the Sn(II) acetate was found at 270~280 °C with an exothermic reaction at 275.5 °C. In addition, a slight endothermic reaction was observed above 280 °C. The exothermic behavior is attributed to decomposition of the organic component contained in the Sn(II) acetate. In Figure 2b, the endothermic behavior after the exothermic reaction is also attributed to the oxidation of the remaining Sn element in order to form SnO_2_ crystals [25]. From the thermal behavior, it is expected that SnO_2_ NPs can be crystallized at below 300 °C with help of the removal of organic components at this temperature. Accordingly, the SnO_2_-Pd NPs were well synthesized with the in situ method followed by heat-treatment at 300 °C.

**Figure 3:**
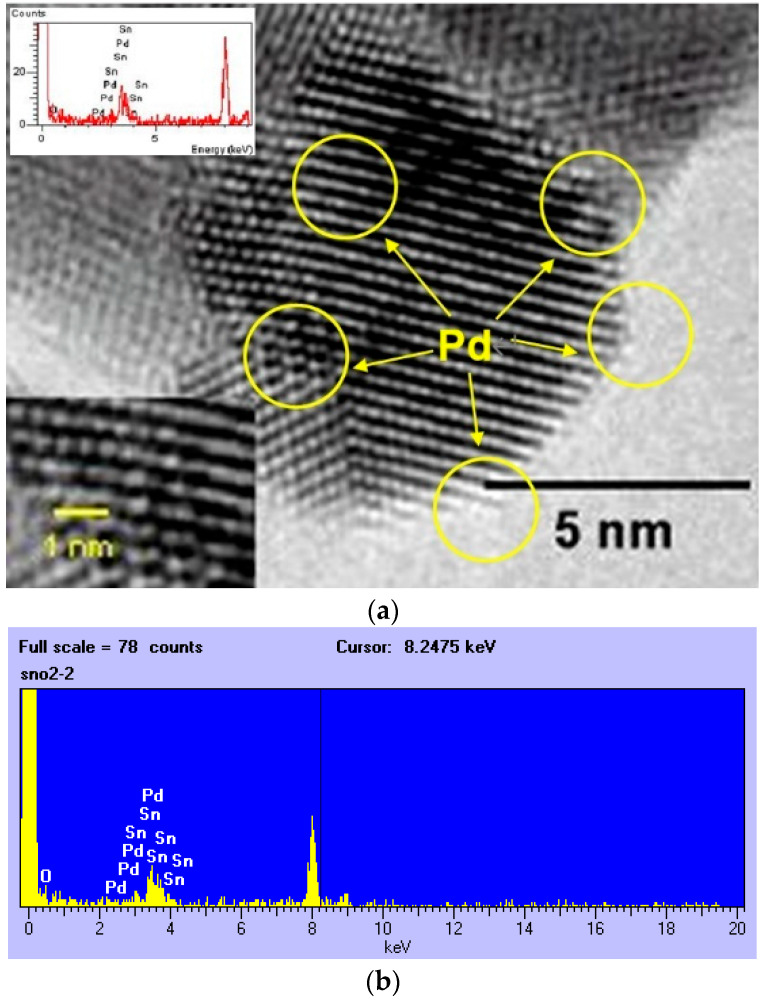
(**a**) HRTEM observation and (**b**) EDS analysis of SnO_2_-Pd NPs synthesized with in situ method (after heat-treatment at 300 °C).

Next, the physical properties of the SnO_2_-Pd NPs synthesized with the in situ method were investigated. Firstly, their particle size was analyzed by using HRTEM. In Figure 3, the size of the synthesized particles is less than 10 nm. In addition, the Pd catalyst is loaded onto the SnO_2_-Pd NPs uniformly. The cluster size of the Pd catalyst is observed to be less than 1 nm and the Pd element was certified by EDS analysis. The BET surface area analysis of the SnO_2_-Pd NPs is 117 m^2^/g, showing very fine NPs.

**Figure 4:**
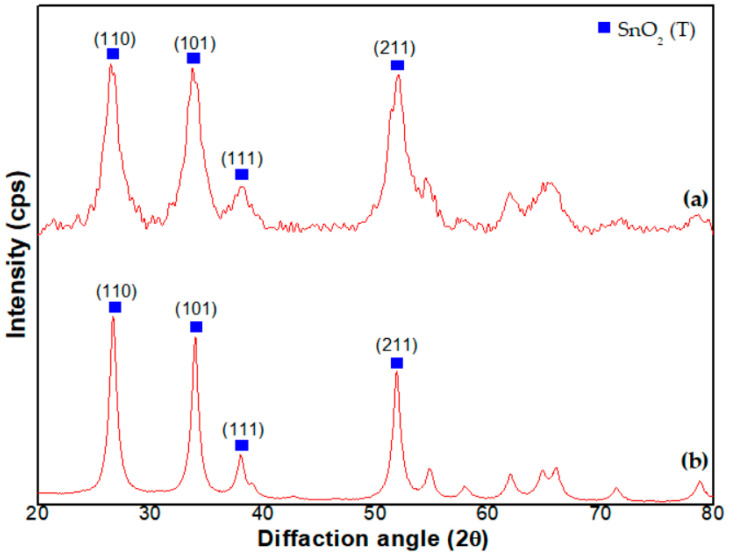
X-ray diffraction peaks of SnO_2_-Pd NPs synthesized with (**a**) in situ method and (**b**) current method, respectively.

The X-ray diffraction pattern, in Figure 4a, also indicates that the Pd catalyst is uniformly distributed in the SnO_2_ NPs. The diffraction pattern is in accordance with that of SnO_2_ NPs synthesized with the current method as shown in Figure 4b, i.e., the NPs are consistently tetragonally structured SnO_2_. Considerable peak shift is not observed in spite of Pd loading, i.e., the lattice parameter of the Pd loaded SnO_2_ NPs is not changed. As a result, a diffracted peak shift is not observed, despite loading at 300 °C.

**Figure 5:**
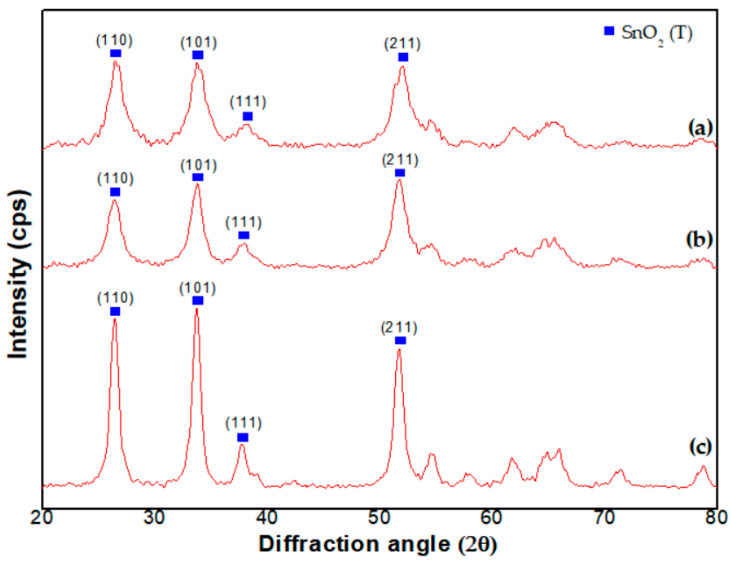
X-ray diffraction peaks of crystal structure of the SnO_2_-Pd NPs after heat-treatment at (**a**) 300 °C, (**b**) 500 °C and (**c**) 700 °C, respectively.

Then, we observed crystal structures of the SnO_2_-Pd NPs after heat-treatment of the precursors at 300 °C, 500 °C, and 700 °C, respectively. The X-ray diffraction patterns are shown in Figure 5a–c, respectively. Tetragonal structured SnO_2_ peaks are observed in all cases. Interestingly, the relative intensities of the X-ray peak diffracted at (110), (101) and (211) planes are dependent upon the temperature, i.e., the preferred orientation of the NPs is strongly associated with the temperature. Especially, (101) peak increases in proportion to the temperature. The increasing peak diffracted at (101) plane of SnO_2_ overlaps with the main X-ray peak of the PdO [26]. Therefore, it is assumed that the increase of the 101) peak is associated with the increase in the main PdO peak at 33.9° as the temperature increases.

**Figure 6:**
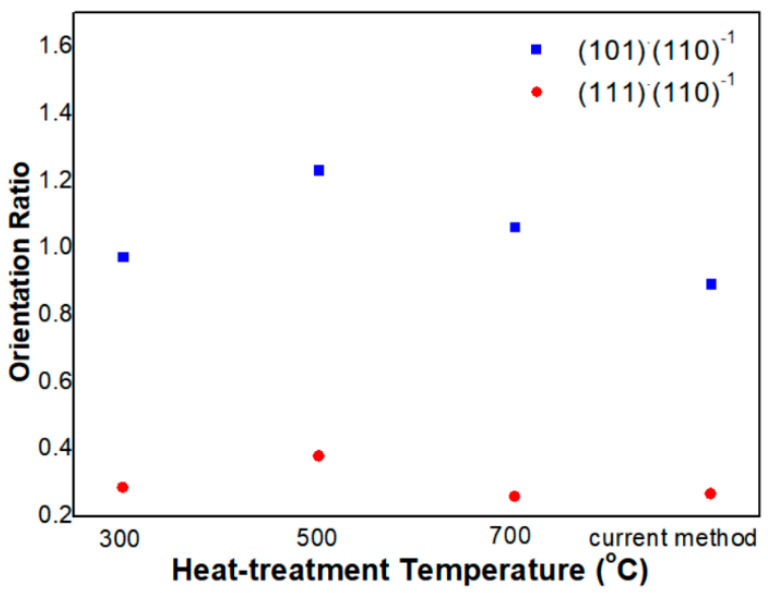
Crystal orientation of the SnO_2_-Pd NPs synthesized under different conditions.

For quantitative analysis, we compared the crystal orientation by calculating the ratio [26], i.e., peak intensity of (110) is divided by (101) and (111), respectively. The smaller value of the orientation ratio means that the crystal structure of (110) orientation is more intensive and closer to the (110) preferred tetragonal structure. As shown in Figure 6, the ratio value of (101)/(101) and (111)/(110) is increased as the temperature increases from 300 °C to 500 °C, meaning that (101) and (111) oriented structures are more dominant than (110) oriented. From the calculation, the orientation when synthesized at 300 °C is mostly coincident with that synthesized with the current method. Therefore, the SnO_2_ NPs synthesized at 300 °C are relatively well crystallized into the tetragonal structure.

### 3.2. Gas Sensing Properties of SnO_2_-Pd NPs Gas Sensitive Thick Films

**Figure 7:**
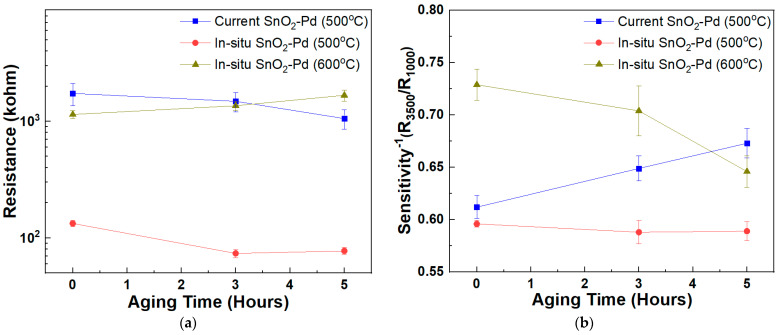
(**a**) Resistances and (**b**) sensitivities of gas sensitive thick films containing SnO_2_-Pd NPs synthesized with current method, in situ method at 500 °C and in situ method at 600 °C, respectively.

Firstly, we compared the sensitivity of the SnO_2_-Pd NPs synthesized with the in situ method to those synthesized with the current one. The thickness of the gas sensitive thick film is c.a. 40 μm. In Figure 7a, the resistances of the two samples are compared. The sample synthesized with the current method shows a higher resistance. Thus, a better sensitivity is expected from the sample synthesized with the current method since, conventionally, the gas sensitive thick film with the higher resistance has shown a superior sensitivity. Unexpectedly, in Figure 7b, the sample synthesized with the in situ method, having the lower resistance, showed superior sensing properties. Moreover, sensitivity of the sample synthesized with the in situ method is very stable after 5 h aging at 400 °C. On the contrary, the sensitivity of the sample synthesized with the current method is degraded according to the aging time at 400 °C.

**Figure 8:**
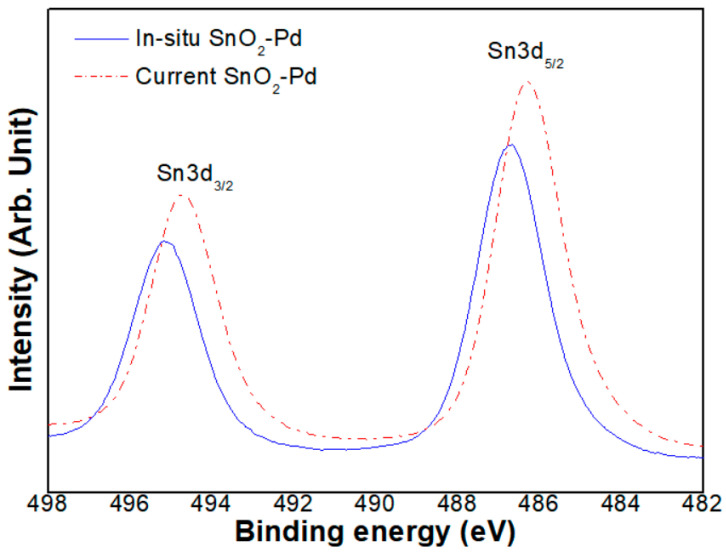
XPS peaks of SnO_2_-Pd NPs synthesized with in situ and current methods, respectively.

It is assumed that this phenomenon is related to the oxygen vacancy in the SnO_2_-Pd NPs. Thus, for a more detailed investigation of the oxygen vacancy, XPS analysis was carried out. As a result, in Figure 8, a difference in binding energy between the two samples was found. That is, the gas sensitive thick film containing SnO_2_-Pd NPs synthesized with the in situ method have a slightly lower binding energy than that synthesized with the current method.

To compare with the XPS result, we heat-treated the gas sensitive thick film at 600 °C. As a result, the resistance increased, accompanied by degradation of the sensitivity. This coincides with the XPS result, and the sample synthesized with the in situ method exhibits enhanced sensing properties. The R_3500_/R_1000_ shows 0.59 and, after aging at 400 °C for 5 h, the sensitivity maintains constant value. Thus, gas sensitive thick film with good and stable sensitivity could be fabricated by applying the SnO_2_-Pd NPs synthesized with the in situ method.

## 4. Discussion

### 4.1. Physical Properties of SnO_2_-Pd NPs

The thermal behaviors are explained by thermal weight change of tin acetate, as shown in Figure 2b. The weight decreased steeply at temperature ranging from 72 °C to 280 °C. Most of the organic components of tin acetate are supposed to be burnt out at this temperature range. From the thermal analysis, all the organic components included in tin oxide seem to be burnt out below 300 °C. Such thermal decomposition under low temperature is enough to suppress the particle growth during the synthesis.

In addition, the X-ray diffraction pattern of SnO_2_ NPs, unchanged after Pd loading, is found in previous literature [27]. This is owing to the uniform distribution of Pd in the SnO_2_ lattice. In the literature, the Sn^4+^ ion is substituted by the loaded Pd^2+^ ion on the SnO_2_ surface [27]. Because of the substitution between the two ions of similar radius, a considerable change in lattice parameter does not occur. In addition, the tendency of (101) and (111) oriented structures, more dominant than (110), is attributed to the loaded Pd catalyst [26], i.e., the PdO (101) reflection is masked with the SnO_2_ (101) peak and leads to an increase of the relative intensity of this peak. The PdO originates from the oxidation of Pd at 500 °C [23]. The ratio is decreased at 700 °C, but the ratio value (101)/(110) is still higher than that synthesized at 300 °C. On the contrary, the value (111)/(110) is lower than that synthesized at this temperature.

### 4.2. Gas Sensing Properties of SnO_2_-Pd NPs Gas Sensitive Thick Films

The difference in the sensitivity between the two samples is owing to the catalytic reaction. That is, in the case of the in situ method sample, uniform catalytic reaction is maintained despite several repetitions of the oxidation–reduction at the Pd catalyst site. However, in the case of current method sample, the reduction does not occur uniformly after oxidation of the Pd catalyst.

These behaviors, along with aging time, suppose that the active cycle of the in situ sample is attributed to oxygen vacancy near the catalyst site [28], i.e., it is probable that the oxygen vacancy acts as an electrical carrier.

The XPS peaks are attributed to a change in the Fermi level to conduction band separation [29]. It is well known that tetragonal SnO_2_ is an n-type semiconductor due to the oxygen vacancies, which act as donors of the conduction electrons. An increase of the oxygen vacancies would imply a shift of Fermi level towards the conduction band, thus decreasing the binding energy. Thus, it is explained that the in situ sample shows superior sensitivity, despite lower resistance than the current sample. It is probable also that the concentration of the oxygen at the Pd catalyst site on the in situ SnO_2_ NPs is lower than that on the current SnO_2_ NPs. In that case, the vacant site lowers the reduction–oxidation reaction energy barrier and, as a result, the more active reaction enhances the sensitivity.

From an assumption based on XPS, the heat-treatment temperature may influence the in situ sample. That is, if the heat-treatment temperature is raised, the vacancy site on the synthesized NPs will be lowered. The resistance of the sample heat-treated at higher temperature may also increase, owing to the increase in oxygen partial pressure at the higher temperature.

## 5. Conclusions

In this study, SnO_2_-Pd NPs were made with an in situ synthesis-loading method followed by heat-treatment at 300 °C. As a result, tetragonal structured SnO_2_-Pd NPs, having an ultrafine size of less than 10 nm and a uniformly distributed Pd catalyst in the SnO_2_ lattice, were well made, and the gas sensitive thick film at a thickness off c. 40 μm was well fabricated by using the NPs. Gas sensing characterization to CH_4_ indicated that the gas sensitivity of the thick film consistency of SnO_2_-Pd NPs synthesized with the in situ synthesis-loading method followed by heat-treatment at 500 °C was enhanced to 0.59. Therefore, the in situ synthesis-loading method is available for synthesis of SnO_2_-Pd NPs for gas sensitive thick film.

## Data Availability

Not applicable.
